# Static Positioning under Tree Canopy Using Low-Cost GNSS Receivers and Adapted RTKLIB Software

**DOI:** 10.3390/s23063136

**Published:** 2023-03-15

**Authors:** Julián Tomaštík, Tim Everett

**Affiliations:** 1 Department of Forest Resources Planning and Informatics, Faculty of Forestry, Technical University in Zvolen, 960 01 Zvolen, Slovakia; 2 RTK Consultants LLC, Niwot, CO 80503, USA

**Keywords:** smartphone, low-cost GNSS receivers, horizontal accuracy, raw GNSS data, vegetation cover, RTKLIB

## Abstract

The decrease in costs and dimensions of GNSS receivers has enabled their adoption for a very wide range of users. Formerly mediocre positioning performance is benefiting from recent technology advances, namely the adoption of multi-constellation, multi-frequency receivers. In our study, we evaluate signal characteristics and horizontal accuracies achievable with two low-cost receivers—a Google Pixel 5 smartphone and a u-Blox ZED F9P standalone receiver. The considered conditions include open area with nearly optimal signal reception, but also locations with differing amounts of tree canopy. GNSS data were acquired using ten 20 min observations under leaf-on and leaf-off conditions. Post-processing in static mode was conducted using the Demo5 fork of the RTKLIB open source software, which is adapted for usage with lower quality measurement data. The F9P receiver provided consistent results with sub-decimeter median horizontal errors even under tree canopy. The errors for the Pixel 5 smartphone were under 0.5 m under open-sky conditions and around 1.5 m under vegetation canopy. The adaptation of the post-processing software to lower quality data was proven crucial, especially for the smartphone. In terms of signal quality (carrier-to-noise density, multipath), the standalone receiver provided significantly better data than the smartphone.

## 1. Introduction

In terms of the use of global navigation satellite systems (GNSS), the last decade has been characterized by a wide-scale expansion to users not formerly specialized in positioning. This is caused mainly by the decreasing costs and dimensions of GNSS receivers. Currently, two main branches in this low-cost GNSS domain can be observed—inclusion of GNSS receivers in mobile devices and the use of standalone receivers.

The localization in smartphones often provides the basis for so-called Location Based Services (e.g., [1]), which link the public services with current or even previous locations of the user. The first GPS-enabled cell phone was introduced in 1999 [2]. Since then, GNSS localization in smartphones has undergone a significant evolution, with the most significant improvements being the multi-constellation and multi-frequency support [3] as well as the ability to record raw GNSS measurements on Android devices [4]. This ability has been continuously analyzed since its implementation. One group of studies focuses on the characteristics of provided GNSS data. These studies include evaluation of the number of tracked satellites [5], code and carrier phase observables [6,7], signal strength/carrier-to-noise density (C/N0) [8,9], multipath [10] and other characteristics. The authors generally agree that the quality of the GNSS raw observables is lower than for higher-grade equipment under comparable conditions. Simple, omnidirectional antennas used in smartphones are often considered the main reason for such an inferiority. The other group of studies is focused on practical applications, taking into account many differing positioning approaches, including single point positioning (SPP) [11,12], precise point positioning (PPP) [13,14], differential GNSS (DGNSS) [15,16], real-time kinematics (RTK) [17,18], post-processed kinematics (PPK) [19] and static (carrier-phase based) positioning [20]. To supplement the low-quality GNSS data from smartphones, authors have recently focused on combinations with other sensors usable for positioning, e.g., inertial measurement units (IMU) [21,22,23]. Other approaches include, e.g., the aid of 3D maps [24] and collaborative smartphone positioning [1]. To achieve relatively stable high-precision results, some problems need to be solved, including lack of phase center offset and variation [6,25], discontinuities in phase observations [26], unaligned chipset initial phase biases [7], and the already mentioned improvement in antenna characteristics [4,27].

Standalone low-cost receivers are being built into user-ready devices on various platforms (pedestrian, automotive, unmanned aerial vehicles, etc.) or combined with other sensors to improve their positioning (e.g., inertial sensors) or other capabilities, including photogrammetry [28] and the Internet of Things [17]. Such devices can provide centimeter-level accuracy, which was recently a privilege of only much more expensive devices. The price of these devices can be in the range of tens to several thousand Euro, mostly depending on particular characteristics (single-, dual-, triple-frequencies, used constellations, etc.) and user friendliness/operation readiness. These receivers often share the problems with smartphone receivers, e.g., they provide lower C/N0 values and worse multipath mitigation than survey-grade equipment [29]. However, taking the receiver–antenna combination into account, some of the problems can be countered using a higher-grade antenna. This on the other side, could negatively affect the cost-effectiveness of the setup. In comparison with smartphone receivers, the precision and accuracy of positioning is significantly higher. Already, first reports on single-system/dual-frequency and dual-system/single-frequency low-cost receivers [30], followed by the analysis of four-systems/single-frequency device [31] suggest results similar to a survey-grade equipment. More recent studies confirm these findings for multi-constellation/dual-frequency low-cost receivers in both kinematic [32,33] and static [34,35] applications.

The majority of studies on the accuracy of low-cost receivers take place under open-sky conditions with ideal reception of satellite signals. This is logical as the new equipment needs to be evaluated under the conditions where the influence of external factors is as low as possible. However, many practical positioning tasks are conducted under conditions with sub-optimal signal reception. Urban and forest (tree vegetation) environments are considered the typical examples of such adverse conditions (e.g., [17,36]). The main constraints here are signal blocking and multipath reflection [36,37]. Combining various techniques, e.g., the already mentioned inertial measurements and 3D maps, is used to mitigate the negative effects. Comparing urban and forest conditions leads to the knowledge that urban conditions with somewhat fewer dynamics and a more regular/schematic structure can be described more directly. LIDAR and image-based maps of cities can be used to identify non-line-of-sight (NLOS) and reflected signals [38,39]. In forests, such approaches can be used to eliminate the influence of relief, which is typically more complicated. However, the high dynamics and variable, complicated structure of the trees’ vegetation defies the enumeration and generalization of its influence. Studies on the influence of canopy openness [12] and differing forest inventory characteristics (diameter, height, species, etc.) [40,41] usually report only low to moderate correlation with the resulting accuracy, even with multi-factorial models. Therefore, in the current state it seems that the way to achieve better positioning results under forest canopies is through the increase of robustness and availability of GNSS measurements, on both the hardware and software sides.

In this contribution, we assess the horizontal accuracy achievable using short-observation, static GNSS measurements provided by a GNSS enabled smartphone and a standalone low-cost GNSS receiver. Special emphasis is given to the influence of tree vegetation with differing degrees of canopy and on dealing with the lower quality signals on the software side. Simultaneous measurements with the tested devices were taken multiple times during the leaf-on and leaf-off seasons to provide information on the repeatability and variability of the results. The basic variability analysis of the carrier-to-noise density C/N0 and multipath, related to the tested devices and conditions, was also conducted.

## 2. Materials and Methods

### 2.1. Data Acquisition

**Figure 1:**
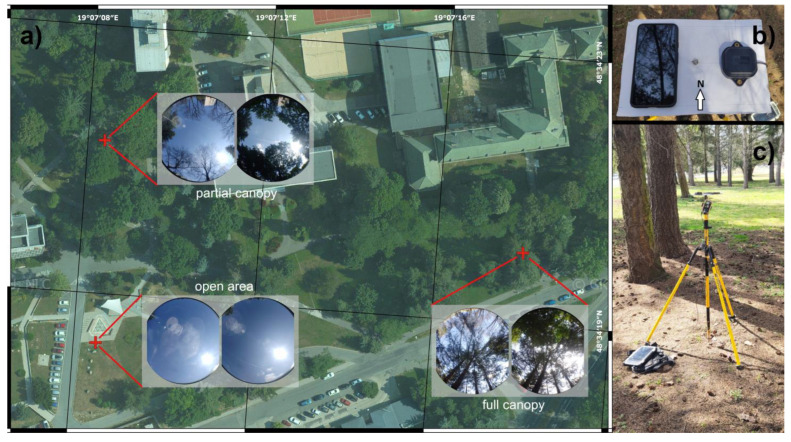
Location and hemispherical images (leaf-off and leaf-on) of test points (**a**), devices’ arrangement with a ground plane (**b**) and overall test setup (**c**) (orthophoto by GKÚ Bratislava, NLC, photo b, c by Ivan Gracík).

Three test points were used to test the capabilities of the low-cost receivers under tree canopy. The first point represents an open area with a minimal number of objects influencing the signal reception up to a 15° elevation angle. The second point is located in a park. It is characterized by a north-facing gap between 45° and 90° elevation angles. In this case, the rest of the sky-view is covered mostly by deciduous trees. The third point is evenly covered by a mix of deciduous and coniferous trees, without any particular gaps in the canopy. Figure 1 shows the location of the points (a), the arrangement of tested devices (b) and the overall test setup (c).

The reference positions were acquired using static GNSS measurements in combination with measurements from a total station. Coordinates in the ETRS89 (ETRF2000, epoch 2008.5) frame are reported in Table 1. This reference frame is used by the national CORS network (Slovak real-time positioning service–SKPOS) to provide correction data.

A Google Pixel 5 smartphone and a u-Blox ZED F9P application board (C099-F9P) were used for the data collection. Both the devices are multi-constellational and dual-frequency. Technical summaries with regard to GNSS capabilities are in Table 2.

Note that in this experiment, only constellations and frequencies that were available from both the rover receiver and the CORS corrections were used in the solutions.

The measurements were conducted simultaneously by both devices which were placed on simple ground planes to reduce the effect of multipath. Before every measurement, the plane was oriented using a compass. The antenna center for the u-Blox receiver (the ANN-MB-00-00 antenna) was shifted 7.5 cm to the east from the reference position, while the center of the smartphone was shifted 6.5 cm to the west. These shifts were considered during the accuracy evaluation.

A total of 20 min of raw measurements were recorded by both devices at each of the test points. Such measurements were repeated ten times under leaf-on conditions (between 28 August and 1 October 2021) and ten times under leaf-off conditions (between 24 February and 4 April 2022). In both periods, the measurements were taken generally between 9:30 and 11:30 GPST. The u-center software (u-Blox AG, Thawil, Switzerland) was used to record the u-Blox ZED F9P data in the proprietary .ubx format. Subsequently, it was converted into the RINEX format using the RTKCONV module of the RTKLIB package. The RINEX format was also used for recording of the smartphone data using the Geo++ Rinex Logger application (Geo++ GmbH, Garbsen, Germany). The data were collected at 1 Hz rate, thus resulting in ~1200 epochs for every set.

### 2.2. Data Processing and Evaluation

The correction data from ZVOL CORS station belonging to the SKPOS network were downloaded for every measurement in the RINEX format. The baseline between the points and the used CORS station was ~2.4 km. These corrections included observations from the GPS, Galileo, GLONASS and Beidou constellations.

The rover data (u-blox F9P and Google Pixel 5) were subsequently post-processed using static positioning mode and the rnx2rtkp.exe executable of RTKLIB software (versions 2.4.3. b34 and Demo5 b34g). All available systems and frequencies were used. Detailed configuration files differences for the smartphone and standalone receiver are available in the dataset [42]. To enable parallel processing (multiple device and canopy conditions at one time) we employed a Python script. After receiving the final coordinates for every solution, these were compared to the reference and differences in Eastings (Δ_E_) and Northings (Δ_N_) were calculated. The horizontal errors were calculated as follows:

$${∆}_{EN}\;=\;\sqrt{{{∆}_{E}}^{2}+{{∆}_{N}}^{2}}$$

These were subsequently used as the main measure for the evaluation of positioning accuracy. Since the distribution of these errors was typically shifted to the left (towards zero), we used medians and percentiles to describe their behavior as well as non-parametric tests (Kruskall–Wallis based) to evaluate the significance of the differences between the groups.

For the signal quality analysis, the mean carrier-to-noise density (C/N0) and multipath descriptors (MP12, MP17 for u-Blox F9P, and MP15 for Google Pixel 5) were calculated using the BKG Ntrip Client [43] and WinTEQC software [44]. The MP12, MP17 and MP15 are linear combinations of pseudorange and carrier phase measurements and their between-frequencies estimates, e.g., MP12 for L1 and L2 [45]. The “7” represents the Galileo E5b frequency, while “5” the E5a. The basic variability analysis was conducted to compare the tested devices, canopy and foliage conditions.

Throughout the analyses, four main variables were considered:

1. Applied device (Google Pixel 5, u-Blox ZED F9P application board)
2. State of vegetation (leaf-on, leaf-off)
3. Level of canopy cover (open area, partial canopy, full canopy)
4. Software and configuration file versions (RTKLIB 2.4.3. b34 with stock configuration file and Demo5 b34g with adapted configuration file)

**Figure 2:**
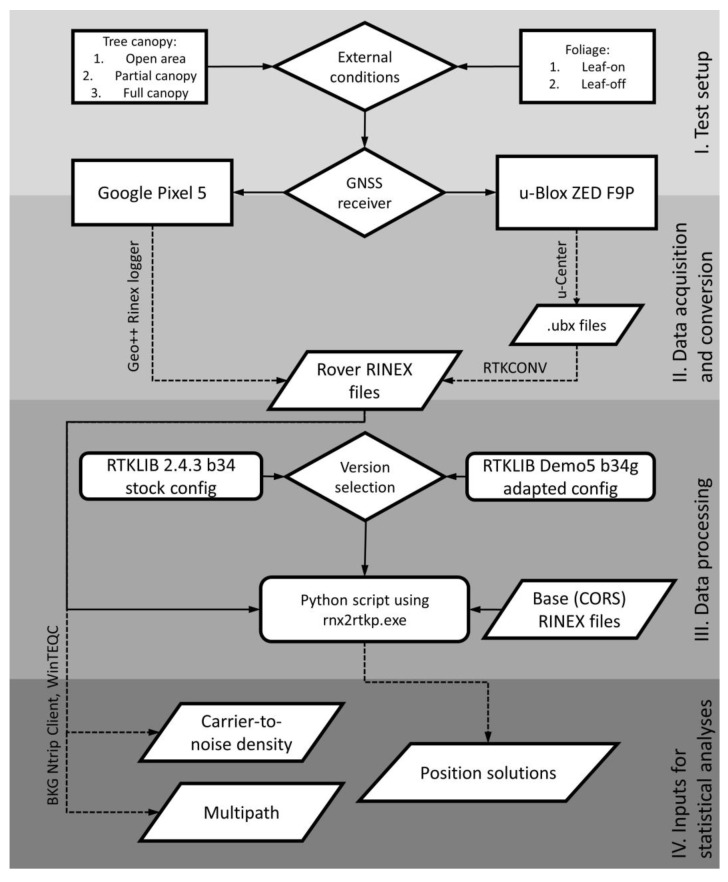
Test inputs, outputs and basic steps of the methodology.

The dataset including the F9P, Pixel 5 and base (ZVOL) GNSS data, configuration files (for the Demo5 b34g version) and the used Python scripts is available via Mendeley Data [42]. A diagram summarizing crucial steps of the test methodology is shown in Figure 2.

### 2.3. Adaptation of RTKLIB Code and Configuration

The RTKLIB package [46], developed by Tomiji Takasu, is one of the most comprehensive open source software packages for GNSS data processing. Based on this code, but with the aim of improving solutions for lower cost receivers and lower quality data, a fork of RTKLIB code named Demo5 has been developed by Tim Everett and is maintained on GitHub [47]. The repository contains code versions as well as changelogs describing the particular changes and enhancements to the code.

An initial baseline set of solutions was generated using the 2.4.3 b34 version of the original RTKLIB code, along with a generic configuration file included with that code and intended for static PPK solutions, with only minor updates made to include all constellations and frequencies. A second set of solutions was then generated using the b34g version of the Demo5 fork of RTKLIB. The configuration file for the F9P solutions in this set was based on a configuration file included with this code that is optimized for the F9P receiver. The configuration file for the Pixel 5 solutions in this set were based on earlier work done with cellphone datasets for the 2021 and 2022 Google Smartphone Decimeter Challenges [19,48]. In both cases, the configuration files were further optimized based on specific environmental conditions of this data and with some iteration of solutions on a subset of the data. A single configuration file was used for all the F9P solutions, and a second configuration file was used for all the Pixel 5 solutions. The purpose of including the baseline data is both to provide a reference to a more commonly used and well-known version of RTKLIB, and to demonstrate the importance of adapting the configuration file for the particular characteristics of the observation data. No attempt was made to distinguish what fraction of the improvement in the solutions was due to the different code versions and what fraction of the improvement was due to the differences in the configuration files. It should be noted that the Demo5 version of the code includes several additional configuration options specifically intended for working with low-quality observation data, thus making a direct allocation of the relative improvements difficult. These additional configuration options include, among other things, more precise handling of outlier thresholds, an additional method of cycle slip detection, and additional retries in the partial ambiguity resolution algorithm.

## 3. Results

### 3.1. Carrier-to-Noise Density

**Figure 3:**
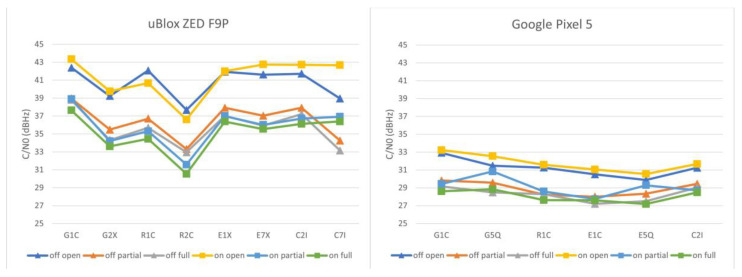
Mean carrier-to-noise density values according to particular tested devices and GNSS signals. Leaf-off values are shown as triangles, while leaf-on values as rectangles.

The comparison of signal strength characteristics for tested devices and conditions is based on the carrier-to-noise density values extracted from raw data. The mean values for particular signals are shown in Figure 3.

**Figure 4:**
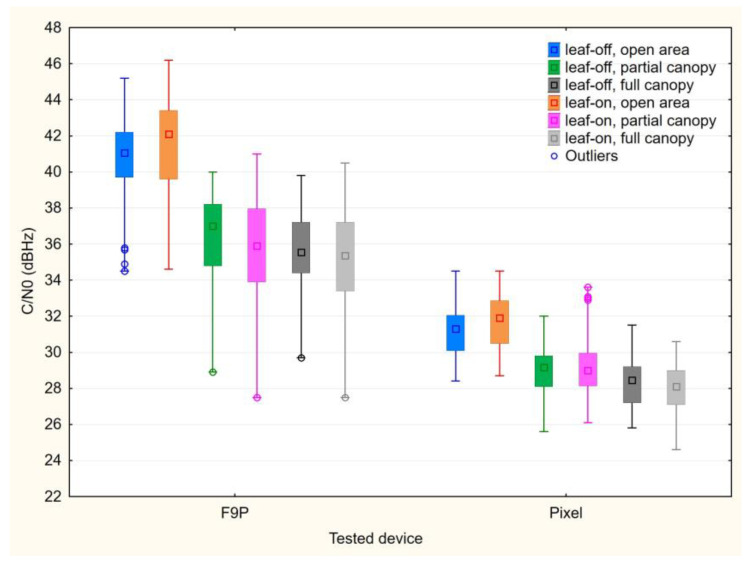
Medians, 25–75% percentiles (boxes), minima and maxima (whiskers) of carrier-to-noise density for tested devices and conditions.

For the u-Blox F9P paired with the ANN-MB-00-00 antenna, the C/N0 averages are ~10–12 dBHz higher than for the Google Pixel 5, taking the comparable signals (G1C, R1C, C2I) into account. The difference between the open area point and the points under tree canopy is clear for the F9P, but apparent also for the Pixel 5. The differences are even more distinct when the values are grouped based on the measurement conditions as shown in Figure 4.

Additionally, the results of Kruskal–Wallis test confirm the significant differences between the tested devices under all considered conditions. For both devices, the differences for the open area point are insignificant under both leaf-off and leaf-on conditions. The open area datasets show significant differences compared to both points under tree canopy, with the exception of the Pixel 5 leaf-off open area set, where the median is higher but not significantly different from the Pixel sets under partial canopy (neither leaf-off nor leaf-on). The comparison of under-canopy subsets leads to the assumption that there is no difference between the partial and full canopy. Furthermore, the differences between leaf-on and leaf-off conditions are insignificant, taking both tested devices and canopy settings into account.

**Figure 5:**
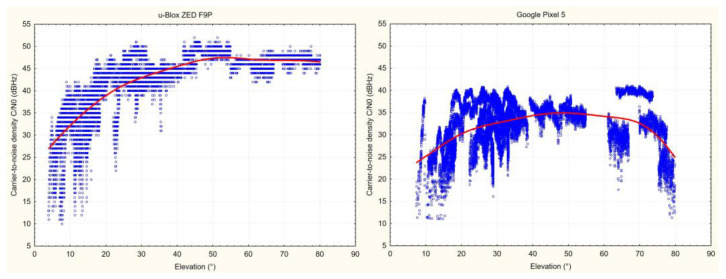
Relation between carrier-to-noise density and elevation for L1 signals and tested devices on the open area point. The trend lines were calculated using the weighted least squares method.

With regard to spatial distribution, Figure 5 shows visible loss in the L1 carrier-to-noise density in higher elevation (>60°) for the Google Pixel 5. 

### 3.2. Multipath

**Figure 6:**
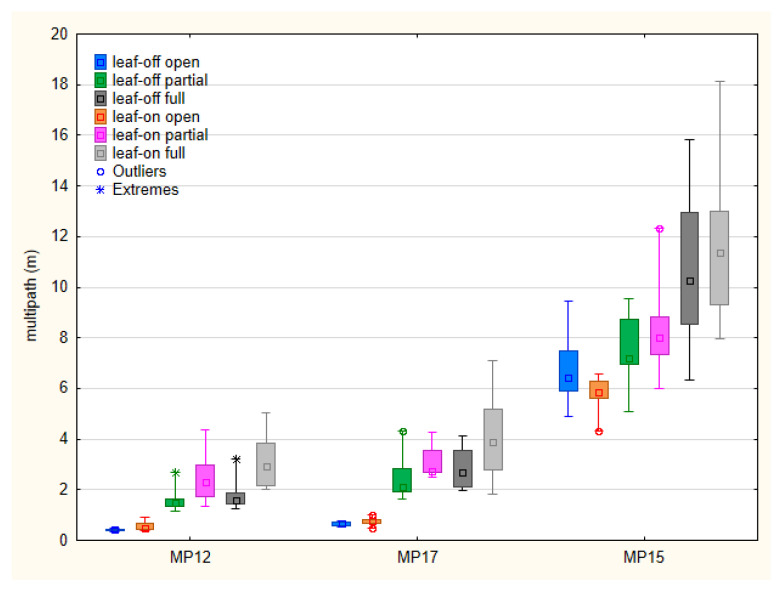
Medians, 25–75% percentiles (boxes), minima and maxima (whiskers) of multipath metrics for tested devices (MP12 and MP17 for u-Blox F9P, MP15 for Google Pixel 5) and conditions.

The multipath was analyzed based on moving average MP12 and MP17 values for the u-Blox F9P and MP15 for the Google Pixel 5. Basic statistical characteristics of these metrics are in Figure 6.

Although the multipath values cannot be directly compared between the devices, the Pixel 5 data appear to be much more influenced by this effect. Median values under open sky conditions are up to 0.79 m for the F9P (MP12 and MP17), while around 6 m for the Pixel 5 (MP15). The medians under vegetation canopy range from 1.48 m to 2.94 m for MP12, from 2.11 m to 3.89 m for MP17 and from 7.22 to 11.36 m for MP15. The comparison between the partial and full canopy shows higher multipath influence for full canopy conditions; however, the difference is not statistically significant in all cases. Taking the leaf-off and leaf-on conditions into account, the MP12 values are significantly higher under leaf-on conditions for both partial and full canopy. For MP17 and MP15, the medians under leaf-on conditions are also higher but the difference cannot be considered statistically significant.

### 3.3. Horizontal Accuracy

**Figure 7:**
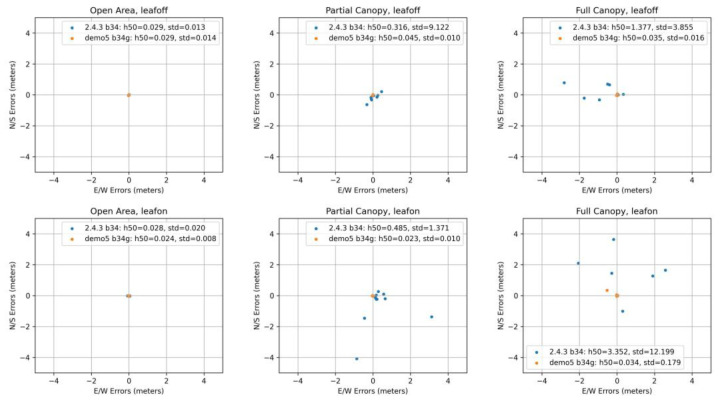
Summary of positioning solutions (including medians and standard deviations) according to canopy and foliage conditions for u-Blox ZED F9P.

**Figure 8:**
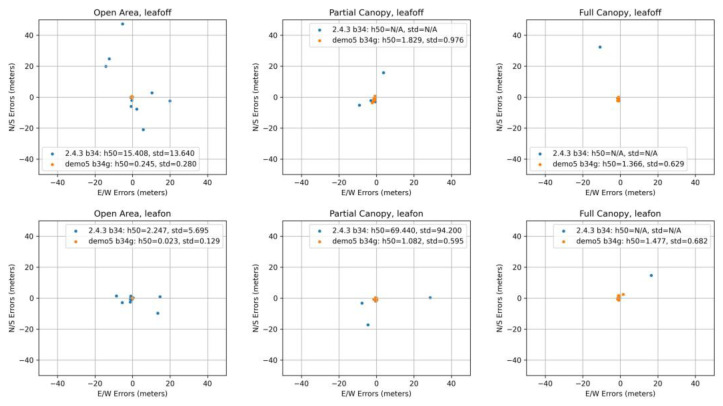
Summary of positioning solutions (including medians and standard deviations) according to canopy and foliage conditions for Google Pixel 5. N/A indicates multiple cases without valid solution.

The summary of positioning solutions is presented in Figure 7 for the u-Blox ZED F9P and in Figure 8 for the Google Pixel 5.

First of all, to avoid unnecessary work in other scales (ranges) and with large outliers, we dealt with the differences between the original RTKLIB 2.4.3 b34 version and the modified Demo5 b34g version. As can be seen in the F9P results, these two versions are fully comparable under the ideal conditions, reaching centimeter-level accuracy. However, the situation changes when moving under tree canopy, where the 2.4.3 solutions reach medians in range from decimeters (partial canopy) to meters (full canopy). A significant increase of variability is also clearly visible. Under the same conditions, the Demo5 b34g solutions still provide centimeter-level accuracy, although a small increase in basic characteristics can be observed. The improved handling of low-quality data by the b34g version is even more visible for the Pixel 5 data. Under open-area conditions, where the b34g solutions were able to provide 0.023 m and 0.245 m medians, the 2.4.3 solutions achieved 2.247 m and 15.408 m values. Under partial and full canopy conditions, the results can be hard to read from the figures because some of the outliers cannot be seen due to maintaining a reasonable scale. Another crucial fact is that the 2.4.3 code was not able to provide any solutions for five full-canopy measurements during the leaf-on season, and five (partial canopy) and three (full canopy) measurements during the leaf-off season. The reasons for these differences in performance are discussed in Section 4.

**Figure 9:**
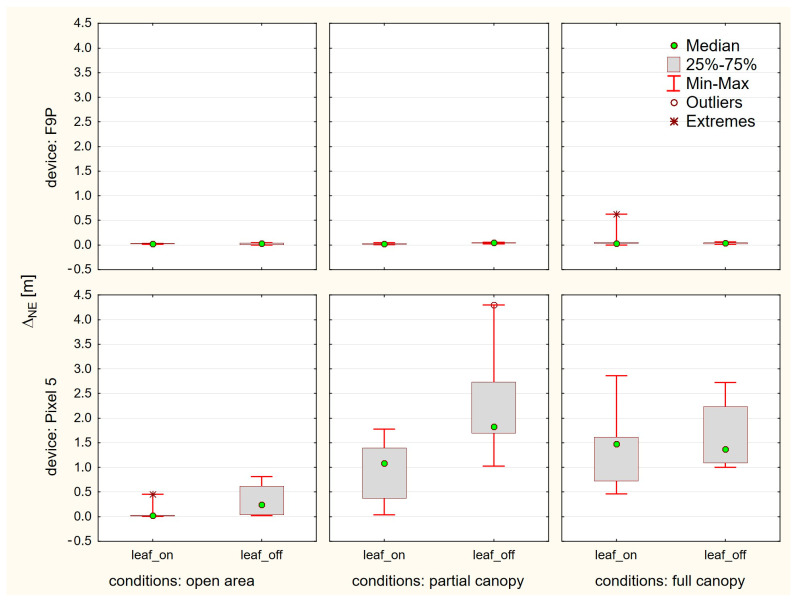
Medians (points), 25–75% percentiles (boxes), minima and maxima (whiskers) of horizontal errors according to device, canopy level and foliage conditions.

For further analyses of other factors, we used only the solutions provided by the Demo5 b34g version. Comparisons regarding canopy level and foliage conditions did not generally affirm some commonly agreed assumptions. The difference between the open-area conditions and conditions under tree canopy (both partial and full) are significantly different only for the Pixel 5. Here the medians increased from 0.023 m (leaf-on) and 0.245 m (leaf-off) to values between 1.082 m and 1.829 m. For the u-Blox F9P, all medians are under five centimeters, thus rendering the differences insignificant. This is even more evident from Figure 9, where we compare medians but also interquartile range and minima and maxima.

The differences between the leaf-on and leaf-off season are insignificant for the F9P. The interquartile ranges for the Pixel 5 are surprisingly higher for the leaf-off conditions. There are a few other phenomena which we do not have a sound explanation for:

1. A single outlier exceeding ten centimeters for the F9P. A horizontal error of 0.629 m is apparent under full canopy, leaf-on conditions.
2. A visible increase of errors for the Pixel 5 on the open-area point during the leaf-off season. The median error is ten times higher than for the leaf-on season. The leaf-off median for Pixel 5 is higher unexpectedly also under partial canopy.
3. Significant bias for all Pixel 5 measurements under tree canopy, shifting them over one meter in the southwest direction. Such a bias is apparent neither on the open-area point nor for the F9P measurements, thus rejecting possible doubts about the reference.

## 4. Discussion

The qualitative differences between the tested devices are apparent already in the carrier-to-noise density values. The increase in the C/N0 of comparable signals is in the range of ~10 dBHz in favor of the u-Blox F9P receiver with the ANN-MB-00-00 antenna. In the present study, the C/N0 values were not compared to a geodetic-grade receiver; however, many authors agree (e.g., [6,26]) that the difference between the survey-grade and smartphone receivers can be the mentioned 10 dB-Hz. The low dependance between the satellite elevation and C/N0 seems to be typical for low-cost receivers/antennas (e.g., [29]). For the Pixel 5, especially, there is a visible occurrence of low-strength signals over higher elevations, which was also reported for other smartphones by Paziewski et al. [9] and Fortunato et al. [49]. The decrease of C/N0 values when comparing the open area with under-canopy conditions was another 4-5 dB-Hz for both tested devices. In contrast to this comparison, the presence of leaves (leaf-on versus leaf-off conditions) did not contribute to the further decrease of signal strength. This can reaffirm the conclusions of Hricko [50] that the main constraint of signal reception in forests is the wooden parts (stems, branches) of the vegetation. On the other hand, the results of the multipath analysis could suggest that, especially for devices able to provide higher quality data (in our case the F9P receiver), the leaves can increase the multipath, and the influence is more visible. For carrier-phase based positioning modes it is important that the signal reception should be continuous. Recent versions of the Android OS provide an option to turn off the so-called “duty cycling”, which is meant to save battery but introduces discontinuities in the data [15,51]. A short check of the data, however, shows that the data from the Pixel 5 smartphone have many more cycle slips than from the F9P receiver (even under optimal conditions and with duty cycling disabled), which could have been reflected in the resulting accuracy. An option to detect cycle slips using a Doppler test, instead of or in addition to using flags in the raw data, is another improvement in the Demo5 code.

Although smartphone antennas are omnidirectional [52], thus providing acceptable signal reception in various poses and uses of the devices, a study by Yong et al. [18] reported that the vertical (upright) position of a smartphone is preferable to the horizontal (lying down) position in terms of RTK positioning accuracy. In our study we used the horizontal position due to a combined test setup (u-Blox F9P and Google Pixel 5) and the use of a ground plane. Overall, the problem of the smartphone “pose” can be of high importance for the practical applications as only limited number of studies deals with GNSS raw data acquired during the most typical types of smartphones uses, i.e., held in hand or pocket [27]. Another fact is that the majority of smartphone applications demand real-time (or near real-time) positioning in dynamic/kinematic applications [18,53]. However, we believe that if stable sub-meter accuracy would be confirmed, even the rapid static methods would find their application for a wider range of users.

Another reason for the sub-optimal performance of smartphone-acquired GNSS data could be software based. The Android API does not directly provide standard GNSS observables, these must be generated using a conversion software. A recent study [54] compared three software capable of converting such data to the common RINEX format. The study also included the Geo++ RINEX logger, which was used in our study. Authors have found discrepancies between generated pseudorange, carrier-phase and Doppler observables. After dealing with the problems, an accuracy of a kinematic test increased by ~26%. The problem can be even more complicated due to the diversity of Android devices as some of the problems were device(chipset)-specific. Such a diversity and fast evolution (although not always in GNSS area) also makes it very challenging to provide timely research with this regard. In our case, the Google Pixel 5 smartphone is already two-generations old.

Besides the factors influencing the acquirement of the GNSS data, the positioning results are also highly dependent on the processing approaches. The RTKLIB package, being open source and highly modifiable, provides an excellent base for various case-dependent adaptations. In our case the main constraint was the significantly lower quality of GNSS data provided by the low-cost receivers, which caused issues when using the original RTKLIB code. We consider the following changes to be crucial for the handling of such data:

1. Validity check dealing with higher residuals. The original 2.4.3. code discarded many data due to low quality (especially under partial and full canopy) to the extent where there was no further solution possible. In the Demo5 code, failing the validity check only creates a warning in the debug file, but does not discard the data.
2. Improved support for L5 frequency. This is important especially for smartphone dual-frequency receivers as they use the L1/L5 combination rather than L1/L2 combination, which is standard for higher-grade dual-frequency receivers. The original code was optimized for the L1/L2 combination, so the Demo5 version improves the utilization of L5 frequency signals.
3. Additional choices and improvements in configuration. Besides the code changes, which in fact represent only a small proportion of the original code, a significant improvement can be achieved by carefully setting up the processing configuration files. In our case, the differences in configurations for the Pixel 5 and F9P receivers were based especially on the quality of the data, which was expected to be lower for the Pixel 5.

Such a high dependency on the proper configuration can lead to “over-configuring” the processing for a particular test case. Therefore, to increase the chance of generalization, such configurations should be tested on differing datasets and kept as simple and comparable as possible. In our case we used one configuration file for all of the Pixel 5 data and one configuration file for all of the F9P data [52,55]. All the aforementioned factors influenced the final positioning accuracy. It was proven that after the adaptation it is possible to use RTKLIB software also with lower quality data from low-cost receivers and achieve reasonable results. For the u-Blox ZED F9P receiver, the results were surprisingly robust and stable even under tree canopy, reaching sub-decimeter accuracy. An improvement in this case could perhaps be achieved by using a calibrated, survey-grade antenna [56]. However, this would negatively influence the cost effectiveness. On the other hand, there is possibly an opportunity to shorten the observation period, thus achieving even better efficiency of surveys. The high accuracy of the F9P receiver was also proven by Wielgocka et al. [34], achieving sub-decimeter accuracy for static, PPP and RTK positioning modes. Hohensinn et al. [32] were able to achieve centimeter-level precision and recommend the receiver for the densification of GNSS networks used for strong-motion seismology and earthquake early warning. A comparison conducted by Hamza et al. [57] resulted in better performance of geodetic instruments, but considering the millimeter-level differences and significantly lower costs, they recommend the low-cost receivers (namely the ZED F9P) also for geodetic applications. Our results suggest that the F9P would be suitable also for cadastral mapping, considering even forested areas, where, e.g., the mean coordinate error must be under 8 cm for establishing new points according to Slovak legislation. The applicability under forest conditions can be further emphasized by the fact that due to higher acreages of forest parcels the points are farther from each other, thus rendering the traditional mapping using polygonal traverses ineffective. The high accuracy combined with the low cost enables also less-traditional applications and combinations, e.g., the direct georeferencing of photogrammetry/lidar data [28,58,59].

The variability of the results for the Google Pixel 5 smartphone is much higher. A significant difference between two subsets measured on an open area point remains unexplained, although even the medians of few decimeters could provide possibilities for many applications. On the other hand, these differences can represent a boundary between applicability/inapplicability in high accuracy tasks, such as the mentioned cadastral mapping. On the same test point, medians of approximately one decimeter were achieved using a Xiaomi Mi8 phone and observation period of 10 min [55]. Retscher and Weigert [16] also evaluated the Pixel 5 phone using a 150 min observation period and multiple positioning modes (SPP, DGNSS, static) under optimal conditions, but due to achieved accuracies they recommend smartphones for GIS applications rather than surveying tasks. Multi-constellation, dual-frequency smartphones seem to provide stable sub-meter accuracies for rapid static measurements under optimal conditions. The requirement is that the smartphone must be able to provide an accumulated delta range (ADR) from which the carrier-phase measurements can be derived [60]. Our results under tree canopy are significantly worse compared with the open area and, in this case, it is clear that the smartphone receiver and antenna combination cannot provide data with quality suitable for high precision positioning. However, even the median errors of ~1.5 m can be considered an improvement over the results of smartphone autonomous positioning and could potentially fulfill the criteria (coordinate error <1.5 m) for mapping of some features (pathways, streams, ridges etc.) for the creation of forestry maps in Slovakia. However, from a practical point of view, the 20 min observation period per point is quite long considering other methods with similar accuracy, for example traditional compass measurement [61] or current mapping using aerial photogrammetry/lidar [62,63]. For ten single- and multi-frequency smartphones, Purfürst [3] reported circular errors probable (CEP_50_, i.e., medians) between 3.28 m and 8.05 m after multiple sessions of 10 min observations under various forest conditions. A CEP of 1.42 m was reported for the Trimble Geo7x geodetic receiver with an external antenna after post-processing. Root mean square horizontal errors of 6.13–12.55 m (leaf-on) and 4.10–11.44 m (leaf-off) were reported for a real-time, single-epoch measurement by Tomaštík et al. [64]. In our case it would be beneficial to identify the reasons causing the unexplained one-meter bias on points under vegetation cover. If it would be possible to remove them, the sub-meter errors would be very promising. However, it is necessary to note that the test points in our experiment were not placed in a forest, where a significantly larger area would have an influence on the GNSS measurements. The actual conditions can be considered urban greenery or the transition between vegetation/urban environment.

Practical evaluation of a GNSS device must consider various characteristics. Besides the measurement accuracy, the user takes cost and work efficiency, user friendliness and other factors into account. In general, smartphones are versatile devices capable of dealing with wide variety of tasks. In the current state, with the focus on high-precision positioning, they suffer from a lack of appropriate hardware (antennas, even external ones) and software (user-friendly applications) capable of providing reasonable results for the most frequent scenarios, i.e., kinematic, real-time (or near real-time) positioning. For standalone low-cost receivers, capabilities for kinematic/real-time applications have been documented, especially under open sky and urban conditions [32,56,65]. The performance under vegetation canopy must be further studied. Considering the smartphone receivers, utilization of raw GNSS data is being studied with aim to provide kinematic/real-time solutions [8,23,49,66]. A periodically held Google Decimeter Challenge [67] provides a very good overview of new approaches focused on post-processing of kinematic smartphone GNSS measurements. The hardware and software diversity of Android devices is also a complicating factor. On the other hand, with the standalone low-cost receivers, the user must consider an investment into the external device but gets a device capable of high-precision positioning in various modes (static, RTK, etc.). The costs can be quite variable. Application/evaluation boards can be purchased for a few hundred Euros but provide limited user friendliness (taking an average user into consideration). The more user-friendly devices, manufactured by multiple companies (e.g., Emlid, Sparkfun) are based on the same GNSS chips but provide better user experience for a higher price. A smartphone is often used as a communication terminal. Regarding accuracy standards, it is not unusual to have varying accuracy requirements for different applications. For example, the California Department of Transportation maintains a standards document with requirements varying from five mm accuracies for primary control monuments, to five cm for topographical features such as signs, and water valves, all the way to ten meters for accident sites [68]. Low-cost technologies such as the u-blox F9P receiver and smartphone GNSS can offer tradeoffs between cost, ease of use and accuracy, potentially providing viable solutions for the many of these different requirement levels. The final decision is on the user; however, in the current state of technology, the standalone low-cost receivers provide high accuracy with a significantly lower effort.

## 5. Conclusions

The results of our study indicate that with low-cost equipment it is possible to achieve reasonable positioning performance even under sub-optimal conditions. However, the differences between the standalone receiver (u-Blox ZED F9P) and the Google Pixel 5 smartphone are significant. With the 20 min observation time and Static positioning mode, the F9P coupled with the ANN-MB-00-00 antenna provided sub-decimeter horizontal errors under all considered conditions. Results achieved by the smartphone were more variable with a visibly negative influence of the tree canopy. This restricts its application to GIS tasks rather than geodetic ones, where the F9P can be considered. These results were achieved using the latest version of the Demo5 fork of the RTKLIB software. The changes in the code and configs, aimed mainly for dealing with lower quality data (originating in both the low-cost hardware and sub-optimal conditions) enabled viable solutions in cases where it was hardly possible with the original code. Thus, the adaptation of processing software was proven crucial for the advanced utilization of the potential of such measurements. The positioning, especially using smartphones, would also benefit from hardware improvements, with antenna improvements being of the highest priority. Overall, the superior costs, wider availability and reasonable accuracy can facilitate the applicability of low-cost receivers also in areas where they were ineffective with more expensive equipment, such as extensive arrays of sensors, IoT, collaborative, crowdsourced acquisition of spatial data and others.

## Figures and Tables

**Table 1:** Reference coordinates of test points.

Point	Easting	Northing
Open area	48.57181624°	19.11913261°
Partial canopy	48.57257093°	19.11909391°
Full canopy	48.57227248°	19.12147211°

**Table 2:** Constellations and frequencies supported by the tested devices.

Device	Constellations	Frequencies
Google Pixel 5	GPS, GLONASS, Galileo, Beidou, QZSS, Navic	L1C/A + L5, L1OF, E1B + E5a, B1I + B2a
u-Blox ZED F9P	GPS, GLONASS, Beidou, Galileo, SBAS	L1C/A + L2C, L1OF + L2OF, E1B + E5b, B1I + B2I

## Data Availability

The RTKLIB Demo5 code is available on Github (https://github.com/rtklibexplorer/RTKLIB, accessed on 14 February 2023). A dataset, inluding GNSS data, RTKLIB config files and Python scripts for batch processing and visualization of results, is available on Mendeley Data.
